# Eschar and neck lymphadenopathy caused by *Francisella tularensis *after a tick bite: a case report

**DOI:** 10.1186/1752-1947-5-108

**Published:** 2011-03-19

**Authors:** Sophie Edouard, Khira Gonin, Yves Turc, Emmanouil Angelakis, Cristina Socolovschi, Didier Raoult

**Affiliations:** 1 Faculté de Médecine et de Pharmacie, URMITE UMR 6236, CNRS-IRD, 27 Bd Jean Moulin 13385 Marseille cedex 05, France; 2 Service de Pédiatrie, Centre hospitalier Laennec, Boulevard Laennec, 60100 Creil, France; 3 Service de Médecine Interne, Centre hospitalier Laennec, boulevard Laennec, 60100 Creil, France

## Abstract

**Introduction:**

In 25 to 35% of cases, the aetiological agent of scalp eschar and neck lymphadenopathy after a tick bite remains undetermined. To date, *Rickettsia slovaca*, *Rickettsia raoultii *and more recently *Bartonella henselae *have been associated with this syndrome.

**Case presentation:**

A four-year-old Caucasian boy was admitted to hospital with fever, vomiting and abdominal pain. On physical examination, an inflammatory and suppurating eschar was seen on the scalp, with multiple enlarged cervical lymph nodes on both sides. Although no tick was found in this scalp lesion, a diagnosis of tick-borne lymphadenopathy was suggested, and explored by serology testing and polymerase chain reaction of a biopsy from the eschar. *Francisella tularensis *DNA was found in the skin biopsy and the serology showed titres consistent with tularaemia.

**Conclusion:**

This is, to the best of our knowledge, the first reported case of scalp eschar and neck lymphadenopathy after tick bite infection caused by *F. tularensis.*

## Introduction

In 1997, Raoult *et al. *described a new tick-borne disease caused by *Rickettsia slovaca *in a patient presenting with a single inoculation lesion of the scalp and cervical lymphadenopathy [1]. In the same year, Lakos reported 27 cases of tick-transmitted infection with similar symptoms: an occipital scalp eschar and painful lymphadenopathy in the region of the tick bite. He named these infections tick-borne lymphadenopathy (TIBOLA) [2]. In 2002, Raoult *et al. *suggested that *R. slovaca *may be a significant cause of TIBOLA [3]. Later, Oteo *et al. *described a similar syndrome in Spain, which they named *Dermacentor*-borne necrosis erythemalymphadenopathy (DEBONEL) [4]. To date, two aetiological agents, *Rickettsia slovaca *and more recently *Rickettsia raoultii *have been unquestionably associated with TIBOLA [5]. Very recently, Angelakis *et al. *described the first cases of TIBOLA caused by *Bartonella henselae *[6] and renamed this syndrome scalp eschar and neck lymphadenopathy after tick bite (SENLAT), to collectively describe this clinical entity. In some cases, the agent causing SENLAT has not been determined [5]. These data indicate collectively that other causative agents of scalp eschars and neck adenopathy remain unknown. We report the first case of SENLAT caused by *Francisella tularensis *after tick bite.

## Case presentation

**Figure 1:**
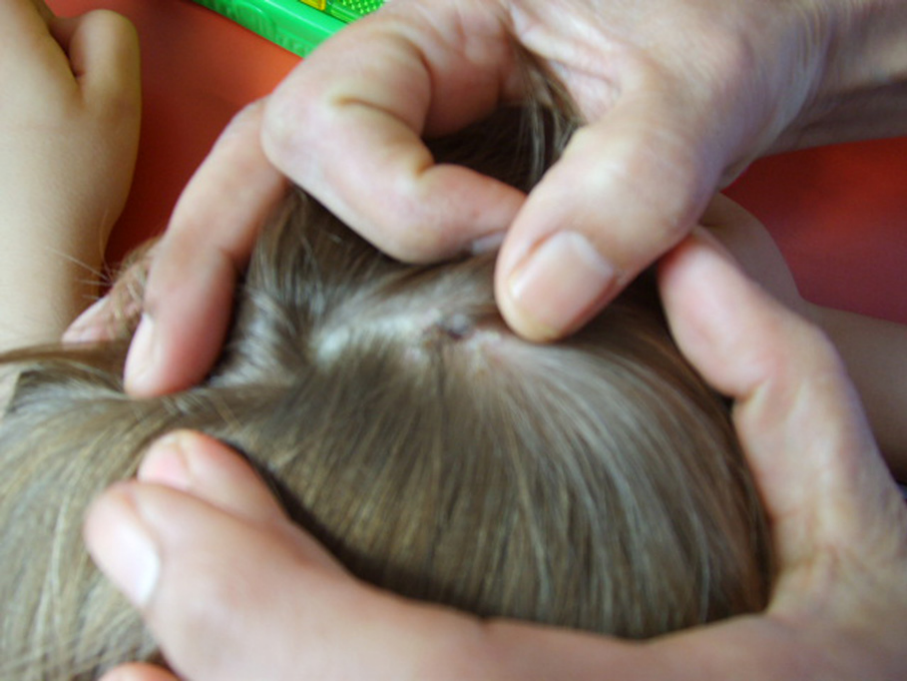
**Eschar on the skin of the scalp**.

A four-year-old Caucasian boy was admitted to the hospital for fever, vomiting and abdominal pain. On physical examination, an inflammatory and suppurating eschar was seen on the scalp (Figure 1) without other specific findings. A scalp lesion that appeared 10 days later was initially diagnosed as folliculitis and treated with pristinamycin. Two days later, the scalp lesions and fever still remained, and with multiple enlarged cervical lymph nodes on both sides. There was tumefaction of the right occiput and stiffness of the right side of the neck. Results of blood cell count and liver enzymes were normal. C-reactive protein, erythrocyte sedimentation rate and alphaglobulin were moderately elevated.

Upon subsequent questioning, the child reported that he lived near a forest and was frequently bitten by ticks, approximately once or twice a week, particularly on the scalp. Although no tick was found in this scalp lesion, a diagnosis of TIBOLA was suggested and explored by serology and PCR of the eschar biopsy.

DNA was extracted from tissue samples (QIAamp Tissue Kit; Qiagen, Hilden, Germany). The eschar biopsy was screened by real-time PCR for *F. tularensis *using primers and probes targeting the *yqaB *gene with PCR carried out in a thermal cycler (LightCycler; Roche Diagnostics GmbH, Germany). The real-time PCR screen was positive for *Francisella tularensis *DNA in the skin biopsy, and negative for *Rickettsia*, *Bartonella *and *Borrelia*. spp., and for *Coxiella burnetii*. This result was confirmed by PCR of the 16S ribosomal RNA gene, which was found to be 99.8% identical to the GenBank sequence for *F. tularensis *(BK006741). The initial serology test, an indirect immunofluorescence assay, was negative. Three weeks later, further serology tests showed titres consistent with tularemia, including titres for IgG of 1: 400 and IgM of 1:100. To confirm the serological result, western blotting was performed on our patient's serum, which showed that *F. tularensis *was the causative agent. Concomitant indirect immunofluorescence assays for *Rickettsia *and *Bartonella *spp., *C. burnetii *and *Borrelia burgdorferi *were negative.

We treated our patient with doxycycline. The eschar and lymphadenopathy decreased after two weeks of treatment.

## Discussion

*F. tularensis *is the causative agent of tularemia, a bacterial zoonotic disease of the northern hemisphere. A wide range of arthropod vectors has been implicated in the transmission of *F. tularensis *between mammalian hosts. These vectors, particularly the *Dermacentor, Amblyomma *and *Ixodes *sp ticks. can also transmit the pathogen to humans [7,8]. *F. tularensis *was first isolated from *Dermacentor andersoni *by Parker in 1924. Tick-borne transmission of tularemia is now known to occur throughout the northern hemisphere, with varying degrees of frequency in different geographic regions [7]. In the USA, Sweden, Finland and Russia, arthropod bites are a common mode of transmission to humans, whereas in central Europe, arthropod-borne tularemia accounts for a much smaller number of human cases [7]. Tularemia can take several forms in humans, depending on the route of entry of the bacterium into the body [8]. After an infected tick bite, ulceroglandular tularaemia is the most common presentation of the disease [9]. Ulceroglandular disease accounts for 60 to 80% of tularaemia cases; affected patients typically present with fever, skin eschar and tender regional lymphadenopathy [10].

## Conclusions

In summary, we report the first case of SENLAT infection caused by *F. tularensis*. Neither scalp eschar nor neck lymphadenopathy due to *F. tularensis *have been described in the literature. Eschar is usually observed on the legs in cases of tick-borne tularemia [10].

SENLAT is a clinical entity most common in young children and women during colder seasons, and it is associated with *R. slovaca, R. raoultii*, *B. henselae*, *B. burgdorferi *and perhaps *F. tularensis *[6]. However, the spectrum of causative agents of SENLAT was likely to be extended because the aetiological agent currently remains undetermined in 25 to 35% of c SENLAT cases [6].

## Competing interests

The authors declare that they have no competing interests.

## Consent

Written informed consent was obtained from the parents of the patient for publication of this case report and any accompanying images. A copy of the written consent is available for review by the Editor-in-Chief of this journal.

## Authors' contributions

SE wrote the initial manuscript, reviewed the patient notes and reviewed the literature on TIBOLA. KG and YT were responsible for patient care. EA and CS assisted in writing the manuscript. DR fully reviewed the final submission. All authors read and approved the final manuscript and participated in this case study.
